# A Genomic and Transcriptomic Study on the DDT-Resistant *Trichoderma hamatum* FBL 587: First Genetic Data into Mycoremediation Strategies for DDT-Polluted Sites

**DOI:** 10.3390/microorganisms9081680

**Published:** 2021-08-07

**Authors:** Domenico Davolos, Fabiana Russo, Loredana Canfora, Eligio Malusà, Małgorzata Tartanus, Ewa Maria Furmanczyk, Andrea Ceci, Oriana Maggi, Anna Maria Persiani

**Affiliations:** 1 Department of Technological Innovations and Safety of Plants, Products and Anthropic Settlements (DIT), INAIL, Research Area, Via R. Ferruzzi 38/40, 00143 Rome, Italy; 2 Department of Environmental Biology, Sapienza University of Rome, P.le A. Moro 5, 00185 Rome, Italy; fabiana.russo1784@gmail.com (F.R.); andrea.ceci@uniroma1.it (A.C.); oriana.maggi@gmail.com (O.M.); annamaria.persiani@uniroma1.it (A.M.P.); 3 Council of Agricultural Research and Economics, Centre for Agriculture and Environment, Via Della Navicella 2/4, 00184 Rome, Italy; loredana.canfora@crea.gov.it; 4 The National Institute of Horticultural Research, ul. Konstytucji 3 Maja 1/3, 96-100 Skierniewice, Poland; eligio.malusa@inhort.pl (E.M.); malgorzata.tartanus@inhort.pl (M.T.); ewa.furmanczyk@inhort.pl (E.M.F.)

**Keywords:** bioremediation, carbohydrate active enzymes (CAZymes), DDT biodegradation, fungal genomics, genome-scale RNA-Seq, Hypocreaceae, secondary metabolites, siderophores, *Trichoderma hamatum*, whole genome sequencing

## Abstract

*Trichoderma hamatum* FBL 587 isolated from DDT-contaminated agricultural soils stands out as a remarkable strain with DDT-resistance and the ability to enhance DDT degradation process in soil. Here, whole genome sequencing and RNA-Seq studies for *T. hamatum* FBL 587 under exposure to DDT were performed. In the 38.9 Mb-genome of *T. hamatum* FBL 587, 10,944 protein-coding genes were predicted and annotated, including those of relevance to mycoremediation such as production of secondary metabolites and siderophores. The genome-scale transcriptional responses of *T. hamatum* FBL 587 to DDT exposure showed 1706 upregulated genes, some of which were putatively involved in the cellular translocation and degradation of DDT. With regards to DDT removal capacity, it was found upregulation of metabolizing enzymes such as P450s, and potentially of downstream DDT-transforming enzymes such as epoxide hydrolases, FAD-dependent monooxygenases, glycosyl- and glutathione-transferases. Based on transcriptional responses, the DDT degradation pathway could include transmembrane transporters of DDT, antioxidant enzymes for oxidative stress due to DDT exposure, as well as lipases and biosurfactants for the enhanced solubility of DDT. Our study provides the first genomic and transcriptomic data on *T. hamatum* FBL 587 under exposure to DDT, which are a base for a better understanding of mycoremediation strategies for DDT-polluted sites.

## 1. Introduction

Anthropogenic halogenic pollutants such as 1,1,1-Trichloro-2,2-bis (4-chlorophenyl) ethane (DDT) and its metabolite products (e.g., 1,1-dichloro-2,2-bis (4-chlorophenyl) ethane (DDD), and 1,1-dichloro-2,2-bis (4-chlorophenyl) ethylene (DDE)) have accumulated in soils and pose significant health and environmental concerns [1,2]. In particular DDT-polluted agricultural sites are of great human health concern due to the environmental persistence of DDT and its metabolite products becoming long-term sources of exposure [3]. 

Mycoremediation of polluted areas is a promising method to remove many hazardous pollutants [4]. The biodegradation of DDT using microfungi has been regarded as an efficient method to remove DDT in DDT-polluted sites without causing significant ecological consequences [5]. 

It has been reported that DDT-resistant microfungi can efficiently degrade DDT, but the metabolic pathways they use are not yet fully understood. Recently, species belonging to *Trichoderma* genus with the ability to metabolize a variety of xenobiotics including DDT and polycyclic aromatic hydrocarbons (PAHs) have been studied [6]. *Trichoderma hamatum* is included among PAH-degrading species, which uses multicopper laccases, ring-cleavage dioxygenases and peroxidases for their degradation [7,8]. The saprotroph *Trichoderma hamatum* FBL 587, collected from DDT-contaminated agricultural soils in Poland, has previously been screened by tolerance in the presence of DDT (1 mg/L) and regarded as a potential autochthonous fungal strain suitable for mycoremediation of DDT-contaminated agricultural soils [5].

It is well known that some filamentous fungi encode genes involved in the cellular translocation of xenobiotics, as well as in the efflux transporters to detoxify xenobiotics; the gene expression products allow their modification (phase I), conjugation (phase II) and secretion (phase III) [9]. Phase I metabolizing enzymes (cytochrome P450s, and monooxygenases) catalyse xenobiotics mainly through hydroxylation/oxidation reactions, phase II conjugating enzymes (e.g., glutathione S-transferase and sulfotransferase) add polar molecules onto xenobiotics, producing water-soluble metabolites, and the phase III secretion system consists of ATP-binding cassette (ABC) and other transmembrane transporters (e.g., major facilitator superfamily (MFS) transporters) that actively export xenobiotic and/or metabolized compounds across the cell membranes [10,11]. Even though the genomic characterization of several strains of *Trichoderma* species [12] and of few strains of *T. hamatum* has been performed [13,14,15], genomic and transcriptomic studies of tolerance and degradation of DDT have not been conducted on *Trichoderma* species. Therefore, the identification of DDT-responsive genes encoded in the *Trichoderma* genome is still lacking. In order to expand our knowledge on the genetic basis for the DDT resistance and removal capability of *T. hamatum* FBL 587, we sequenced and analysed its genome to provide a foundation of comprehensive understanding regarding the application of this strain for mycoremediation strategies of DDT-polluted sites.

Here we present the 38.9 Mb-genome sequence of *T. hamatum* FBL 587. We performed an analysis of 10,944 protein-coding genes that were predicted and annotated, focusing on those associated to carbohydrate-active enzymes (CAZymes), secondary metabolites (SMs), and siderophores, all of relevance to mycoremediation. In the genome we also investigated genetic repertoires potentially involved in the resistance and metabolism of DDT. Moreover, transcriptomic analysis was performed to observe differential expression of genes by DDT (10 mg/L). The genome-scale RNA-Seq study performed on exposure of *T. hamatum* FBL 587 to DDT highlights genetic features that shed a light on the DDT tolerance and removal by this strain. Finally, the DDT-degradation performance of the strain was tested in a pot experiment.

## 2. Materials and Methods

### 2.1. DDT Degradation Process in Soil by T. hamatum FBL 587

A pot experiment was performed to assess the potential additive impact of *Trichoderma hamatum* (Bonord.) Bainier FBL 587 application on DDT phytoremediation capacity of two different *Cucurbita pepo* accessions, a species with well described and documented potential in bioremediation [16]. Soil originating from a field that in several preliminary analyses did not show any contamination with DDT was spiked in with analytical grade p,p′-DDT (Sigma Aldrich). A stock solution of DDT in acetone was mixed with water (3:97 *v*/*v*) to prepare the working solution applied to the soils. The DDT solution was uniformly sprayed using a hand sprayer onto 20 L of homogenised soil arranged as a 2-cm-thick layer over a plastic film, in a quantity necessary to deliver 0.125 mg of analytical grade p,p′-DDT per litre of soil. However, the initial concentration of DDT in soil was determined. The contamination procedure was performed individually for the soil used for growing each selected accession for the bioremediation experiments. Two *C. pepo* accessions were selected for this study: AMES 26607 and PI 614701 grown from seeds obtained from USDA Germplasm Repository. The seeds were germinated in Petri dishes under controlled conditions (25 °C and 80% RH) and after about two weeks, on 27 July 2019 they were planted in artificially DDT-contaminated soil not sterilized. On 30 August the plants were watered with 400 mL/pot of *T. hamatum* FBL 587 (4 × 10^4^ spores/mL), precultured on Malt extract agar (MEA) at 25 °C in the dark. Plants treated with water were used as control. The experiment, done in triplicate (3 pots each having 2 plants), was ended on 9 October. The plants were separated from soil and each plant fraction (root, aboveground and sometimes fruits) together with remaining soil were analysed for DDT residues. DDT and its metabolites were determined as described elsewhere [3]. 

### 2.2. Genomic DNA Extraction, Whole Genome Sequencing and Bioinformatics Analyses

Genomic DNA was extracted from mycelial samples of *T. hamatum* FBL 587 using the QIAamp DNA Microbiome Kit (Qiagen, Hilden, Germany) and was used for Illumina library preparation. DNA quality and concentration were assessed with Nanodrop 2000 (Thermo Fisher Scientific, Wilmington, DE, USA). The paired-end genomic libraries (2 × 250 bp) were built using the MiSeq v.3 reagents (600 cycles) and sequenced at Eurofins Genoma Group (Rome, Italy).

Reads quality was assessed with fastQC v.0.11.9 [17], and adapters removal was performed with Trimmomatic v.0.38 [18]. The filtered reads were de novo assembled using SPAdes v. 3.11 [19] with default parameters, in combination with BayesHammer (distributed with SPAdes latest version) [20] for reads error correction. Nuclear genome statistics were calculated using QUAST v.4.5 [21]. Ribosomal RNA (rRNA) and transfer-RNA (tRNA) were predicted with RNAmmer v.1.2 [22] and tRNAscan-SE v. 2.0 [23], respectively. Simple repeats and low complexity DNA sequences were detected by the program RepeatMasker with the search engine HMMER on *Trichoderma* [24].

The quality assessment for genome assembly, the gene structure annotations, and the completeness of the assembly genome was assessed using GenomeQC [25]. De novo gene prediction was performed with Augustus v.2.5.5 [26] using *Fusarium graminearum* as the training species. Functional annotation was performed with PANNZER2 [27]. Protein function prediction assigned Gene Ontology (GO) terms to proteins, specifying molecular functions, involvement in biological processes and subcellular localizations. For the enzymes obtained from sequenced genomes, protein IDs (ProtID) are used as gene identifiers, while for other enzymes, GenBank accession number (GbID) are provided.

Carbohydrate-Active enZYmes (CAZymes; http://www.cazy.org, accessed on 1 June 2021) for complex carbohydrate metabolism were identified with the dbCAN2 [28], which integrates three state-of-the-art tools for CAZome (all CAZymes of a genome) annotation: (i) HMMER search (*E*-Value < 1 × 10^−15^, coverage > 0.35) against the dbCAN HMM (Hidden Markov Model) database; (ii) DIAMOND search (*E*-Value < 1 × 10^−102^) against the CAZy pre-annotated CAZyme sequence database and (iii) Hotpep search (frequency > 2.6, hits > 6) against the conserved CAZyme short peptide database.

Secondary metabolites (SMs) biosynthesis gene clusters (BGCs) identification and characterization were performed with antiSMASH v. 6.0.0 [29]. AntiSMASH detects genome regions containing biosynthetic gene clusters based on conserved biosynthetic enzymes from different biosynthetic pathways. The antiSMASH database (available at https://antismashdb.secondarymetabolites.org/, accessed on 1 June 2021) contains 177 fungal representative high-quality genomes. The identified regions were compared against a dataset of manually curated biosynthetic gene clusters with known products from the MIBiG reference database. Four *Trichoderma* genomes of the section *Trichoderma* (see [15]) and the genome of *T. lixii* MUT3171 [4] of the clade *Harzianum*/*Virens* (see [15]) are included in the antiSMASH analysis. *Trichoderma atroviride* IMI 206040 (ATCC 20476) is the representative *Trichoderma* high-quality genome in the antiSMASH database (https://antismashdb.secondarymetabolites.org, accessed on 1 June 2021; [30]), and *T. lixii* MUT3171 was chosen to represent the outgroup species.

### 2.3. RNA Extraction and mRNA Sequencing

Five millilitres of spore suspension (5 × 10^6^ conidia/mL) from *T. hamatum* FBL 587 were added to 250 mL flask with sterilized 95 mL of Potato Dextrose Broth (PDB) and incubated for 48 h at 25 °C in the dark on a rotary shaker (150 rpm, ASAL 711/D). Then, DDT was added to each 250 mL flask to get the final concentration of 10 mg/L, except in the control. After the DDT addition, both control and treatment, prepared in triplicate, were incubated for 48 h at 25 °C in the dark. 

The RNA sequencing experiment was performed on six samples. RNA-Seq studies were performed on three replicates for both control and treatment (exposure to DDT at 10 mg/L for 48 h). For transcriptomic analysis, total RNA was extracted from fresh 100 mg of fungal biomass from 2 days of culture. The grown mycelia prepared in triplicate were harvested from the culture media by centrifugation and washed twice with 50 mL distilled water. The mycelium pellet was immediately homogenized using a Powerlyzer^®^ 24 homogenizer (QIAGEN, Germantown, MD, USA) and total RNA was isolated using TRI Reagent (Sigma-Aldrich, St. Louis, MO, USA). Briefly, 1 mL of TRI Reagent was added to 100 mg of *T. hamatum* FBL 587 in RNase-free and DNase-free microtubes and shaken vigorously for 45 s in Powerlyzer^®^ 24 homogenizer, (QIAGEN). After homogenizing the samples with TRI Reagent, the mixture was centrifuged at 12,000× *g* for 10 min at 4 °C to remove the insoluble material. The supernatant containing RNA and proteins was transferred to a new microtube, in which cold absolute chloroform (0.2 mL per mL of TRI Reagent used) was added. The homogenate was incubated at room temperature for 15 min and then centrifuged at 12,000× *g* for 15 min at 4 °C to separate into a clear upper aqueous layer (containing RNA), an interphase, and a red lower organic layer (containing the DNA and proteins). Total RNA was precipitated from the aqueous layer with 1 mL of absolute 2-propanol (>99.5%, molecular grade; Sigma-Aldrich) per mL of TRI Reagent used for 10 min at room temperature. The mixture was centrifuged at 12,000× *g* for 10 min at 4 °C to allow the precipitation of the RNA pellets. The precipitated RNA was washed twice with 1 mL of 75% ethanol to remove impurities, vortexed and centrifuged at 7500× *g*, at 4 °C for 5 min, and then re-suspended in DEPC-treated MiniQuantum (deionised) water and stored at −80 °C for further use. 

Purity and quality of RNA was determined at Genomix4Life (Salerno, Italy) via NanoDrop spectrophotometer and Agilent Bioanalyzer 2100 (Agilent Technologies, Inc., Santa Clara, CA, USA), and a minimal RNA integrity number (RIN) of 7.0 was required to continue with the analysis. An RNA library was constructed using TruSeq Stranded mRNA kit (IL, San Diego, CA, USA) according to manufacturer’s instructions. The sequencing of the RNA library was carried out in NextSeq 500 Illumina system using a single-end approach (1 × 75 bp) producing 27.5 M of reads per sample, on average (Supplementary Table S1).

### 2.4. Transcriptome Profiling with RNA-Seq Approach

A quality check was performed on the raw sequencing data, removing low quality portions while preserving the longest high quality part of NGS reads (Supplementary Table S1); the minimum length was set to 35 bp and the quality score to 25 by means of the software BBDuk (https://jgi.doe.gov/data-and-tools/bbtools/, accessed on 1 June 2021).

The alignment of the high quality reads against the *T. hamatum* FBL 587 genome (present study) was performed with STAR aligner version 2.5.2b [31]. 

FeatureCounts version 1.5.1 [32] was used to calculate gene expression values as raw fragment counts. Normalization was applied to the raw fragment counts by using the Trimmed Mean of *M*-values (TMM; Supplementary Table S2: TMM_table.xlsx) and Reads Per Kilobase Million (RPKM; Supplementary Table S3: RPKM_table.xlsx). All the statistical analyses were performed with R (v 4.1) with the packages HTSFilter and edgeR [33].

The HTSFilter package, which implements a filtering procedure for replicated transcriptome sequencing data based on a Jaccard similarity index, was used to remove genes either not-expressed or showing high variety in the transcript expression among replicates (Supplementary Figure S1). The edgeR determines differential expression using empirical Bayes estimation and exact tests based on a negative binomial model.

To check if the RNA-Seq data from different conditions correlate to each other, a principal component analysis (PCA) was performed. The analysis of the RNA-Seq read counts to each annotated locus was performed in order to identify differential expression of genes by exposure to DDT among samples, i.e., with a FDR (False Discovery Rate (Benjamini-Hochberg correction)) ≤ 0.05. Among the significantly regulated genes (FDR ≤ 0.05), log2FC (fold change relative to control) ≥ 1 indicated upregulated genes while log2FC ≤ 1 indicated downregulated genes.

### 2.5. Accession Numbers of the Genome and Transcriptome Sequences

The whole genome sequence project of *T. hamatum* FBL 587 has been deposited at DDBJ/ENA/GenBank under the accession SEIV00000000 (BioProject PRJNA513966, BioSample SAMN10717514; Sequence Read Archive (SRA) SRS5810831). The version described in this paper is version SEIV01000000.

The RNA-sequencing data have been deposited in NCBI SRA under the following IDs: SRR14056734-5-6 for DDT-treatment samples, and SRR14056737-8-9 for control samples (BioProject PRJNA513966).

## 3. Results

### 3.1. DDT Degradation Process in Soil by T. hamatum FBL 587

**Figure 1:**
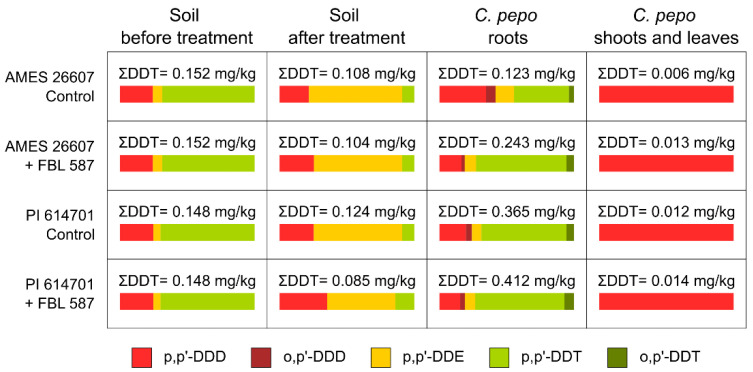
Effect of the soil inoculation with *T. hamatum* FBL 587 on the DDT degradation in soil and phytoremediation capacity of two *C. pepo* accessions. The percentage of each DDT metabolite or residue detected in each fraction and treatment are represented by color bars. The total concentration of DDT metabolites and isomers (ΣDDT) is indicated for each treatment.

The initial concentration of DDT in the artificially contaminated soil ranged between 0.148 and 0.152 mg/kg. The proportion among DDT metabolites in the soil before the treatment was similar among the tested variants and the contamination was determined mainly by the p,p′-DDT (68–69% of total). In all cases, a decrease in the ΣDDT (sum of all metabolites and isomers) concentration was observed at the end of the experiment: it ranged between 57% to 84% of the initial contamination, with the most promising results for combined treatment with *C. pepo* PI 614701 and *T. hamatum* FBL 587 (Figure 1). Interestingly, the main metabolite detected in soil after the treatment, regardless of the studied variant, was p,p′-DDE. Uptake of DDT by *C. pepo* was confirmed by the determination of residues in both root and above-ground parts of the plant, but without translocation to the edible parts (for detailed result in Supplementary Table S4). Soil inoculation with *T. hamatum* FBL 587 decreased the overall DDT concentration in soil and induced an increased accumulation of DDT in roots of both *C. pepo* accessions. However, soil inoculation with *T. hamatum* FBL 587 did not affect the capacity of the plant to translocate the DDT to the above-ground tissues.

### 3.2. Genome Properties of T. hamatum FBL 587

The draft genome sequence of FBL 587 was 38.96 Mbp long with a GC content of 48.54%. In total, 10,994 protein-coding genes were predicted in the genome, of which 8564 (79%) were assigned to different GO terms. The genome contains single copies of 18S and 28S rRNAs, 50 8S rRNAs, 230 tRNAs and 14 pseudogenes (Table 1). We found 1.2% repetitive elements for FBL 587, with the most contribution due to simple repeats (Table 1). As shown in Table 1, the genome was 98.5% complete as predicted by the benchmarking universal single-copy ortholog (BUSCO) in GenomeQC [25]. Among the BUSCO genes, 98.5% (including 0.6% duplicated genes) were found in the assembly, as calculated with BUSCO version 3.0.2 using the trained AUGUSTUS species: *Fusarium graminearum* and “Pezizomycotina_odb9” data set. More statistics on nuclear genome assembly and annotation are provided in Table 1.

### 3.3. Prediction of Genes Encoding for CAZymes, SMs and Siderophores

The genomic analysis of *T. hamatum* FBL 587 revealed the presence of a broad range of genes encoding for CAZymes, proteins involved in the production of SMs and siderophores, all of possible relevance to mycoremediation. 

**Figure 2:**
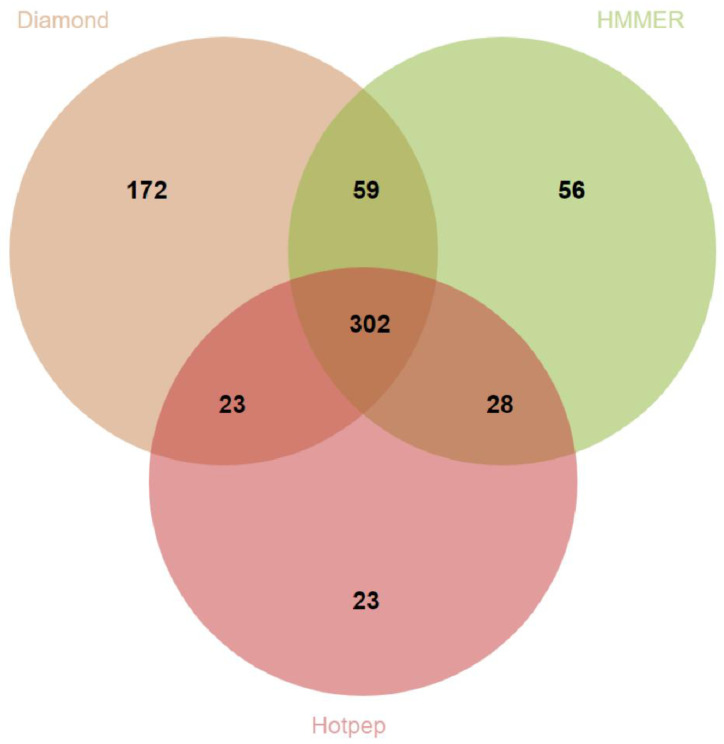
Venn diagram showing overlaps among the annotation results in *T. hamatum* FBL 587 for multi-domain CAZymes of three different tools: (1) HMMER, (2) DIAMOND, (3) Hotpep (see text).

A genomic repository of CAZymes allowed to analyze the pattern of carbohydrate metabolism. In *T. hamatum* FBL 587 genome, selecting HMMER with dbCAN domain assignment we identified a total of 445 CAZymes (302 when considering all three different tools; Figure 2), including 66 auxiliary activities (AA), 4 carbohydrate-binding modules (CBM), 20 carbohydrate esterases (CE), 249 glycoside hydrolases (GH), 94 glycosyltransferases (GT), and 11 polysaccharide lyases (PL) (a full account of all the CAZymes is presented in Supplementary Table S5). 

With respect to genes involved in the formation of SMs, a class of genes of relevance to mycoremediation, in FBL 587 a genomic repository of potential SMs driven by non-ribosomal peptide synthases (NRPS), polyketide synthases (PKS), hybrids NRPS-PKS and terpene was surveyed. Using the tool antiSMASH, version 6.0.0 (see [29]), we identified a relatively few number (27) of candidate BGCs for the production of SMs in *T. hamatum* FBL 587 (Table 2; Supplementary Table S6). Most of the antiSMASH-6.0-detected gene clusters cannot be annotated as their composition is not similar to any known fungal BGC (Supplementary Table S6). In *T. hamatum* FBL 587, we identified 8 PKS, 4 NRPS, 4 NRPS-PKS hybrids, and 6 terpene gene clusters (Table 2). The SM gene clusters found in the other *Trichoderma* species included in the paper for the antiSMASH-6.0 based-analysis are reported in Table 2 and Supplementary Tables S7–S11.

In the genome of *T. hamatum* FBL 587 (node_397_length_30509_cov_30.411329), *g8761* was identified using antiSMASH version 6.0.0 as the gene that shares homology with the T1PKS gene belonging to a known gene cluster, MIBiG accession BGC0001284, in *Parastagonospora nodorum* required for the production of alternariol (Supplementary Table S6). Alternariol is a compound that showed significant radical-scavenging (antioxidant) activity [36]. Our antiSMASH-based analysis found this putative T1PKS gene cluster for alternariol biosynthesis also in *T. hamatum* GD12, as reported in Supplementary Table S10. 

Our antiSMASH-6.0-based analysis also demonstrated that in *T. hamatum* FBL 587 a BGC containing a squalene synthase (ProtID g4482) showed a composition similar (40%) to a known cluster (MIBiG accession BGC0001839) for biosynthesis of squalestatin S1 or squalestatin analogues [37], as reported in Supplementary Table S6. This putative squalestatin S1 gene cluster was also identified in the genome of *T. hamatum* GD12, *T. atroviride* IMI 206040, *T. gamsii* T6085, *T. asperellum* CBS 433.97, and *T. lixii* MUT3171 (Supplementary Tables S7–S11). 

Moreover, in *T. hamatum* FBL 587 (node_21_length_162673_cov_29.833026), *g1187* encoding a member of the terpene cyclase/mutase family was identified through sequence analysis using antiSMASH v. 6.0.0 as the gene that shares homology (48% at amino acid level) with a gene encoding for oxidosqualene cyclase (GbID ACF70484; GbID HYPSUDRAFT_48575) belonging to the antitumor clavaric acid biosynthetic gene cluster, MIBiG accession BGC0001248, of the basidiomycete *Hypholoma sublateritium* [38,39], as reported in (Supplementary Table S6). The antiSMASH-based analysis identified this putative gene cluster in the other *Trichoderma* genomes of the section *Trichoderma* included in this paper, namely *T. hamatum* GD12, *T. gamsii* T6085, *T. asperellum* CBS 433.97, and *T. atroviride* IMI 206040, while it was not found in the outgroup species *T. lixii* MUT3171, as shown in Supplementary Tables S7–S11.

**Figure 3:**
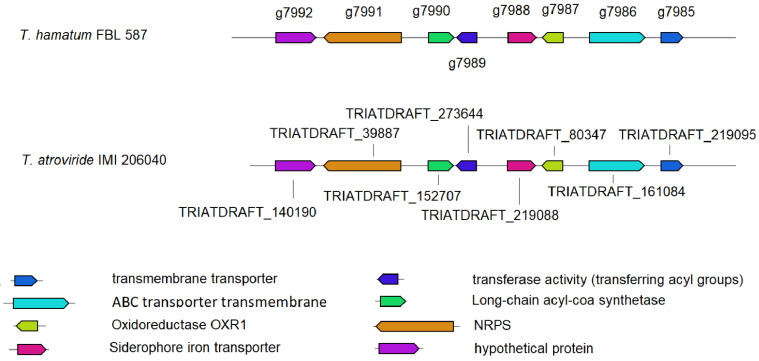
The putative extracellular siderophore dimethylcoprogen gene cluster identified using the tool antiSMASH, version 6.0.0 (see [29]) in the genome of *T. hamatum* FBL 587. For clarity, only the orthologue gene cluster from *T. atroviride* IMI 206040 (representative *Trichoderma* high-quality genome in the antiSMASH database; https://antismashdb.secondarymetabolites.org, accessed on 1 June 2021; [30]) was used for comparison. In *T. hamatum* FBL 587, the candidate BGC genes were assembled to a putative dimethylcoprogen BGC (node_221_length_57612_cov_30.100447, 29,375 nt, start: 28237, end: 57612). The sequence analysis revealed the presence of eight open reading frames, *g7985*–*g7992*, of which at least 5 genes are highly likely to be involved in dimethylcoprogen biosynthesis. The gene *g7985* encodes a putative transmembrane transporter, *g7986* encodes for a homologue to ABC transmembrane transporter, *g7987* encodes for a homologue to oxidoreductase, g7988 encodes for a homologue to the siderophore iron transporter, *g7989* encodes for a homologue to acetyltransferase activity, *g7990* encodes for a homologue to long-chain acyl-CoA synthetase, *g7991* encodes for a NRPS protein, and *g7992* encodes for a hypothetical protein of unknown function. Sequence comparison using BLASTN analysis indicated that the NRPS cluster of *T. atroviride* IMI 206040, locus: NW_014013633, 430,88 bp, start: 76225, end: 119313, showed substantial similarity (identity at nucleotide level of 86.35%, cover: 87%) in relation to that of *T. hamatum* FBL 587. The NRPS cluster of *T. atroviride* IMI 206040 putatively consisted of eight genes. The gene locus_tag TRIATDRAFT_219095 encodes for a protein with predicted transmembrane domains; the gene locus_tag TRIATDRAFT_161084 encodes for predicted MDR-type topology of transmembrane domains and nucleotide-binding folds (TMD6-NBF)2; the gene locus_tag TRIATDRAFT_80347 encodes for a hypothetical protein; the gene locus_tag TRIATDRAFT_219088 encodes for hypothetical protein with transmembrane domains; the gene locus_tag TRIATDRAFT_273644 encodes for a putative siderophore biosynthesis protein; the gene locus_tag TRIATDRAFT_152707 encodes for a NRPS-like protein, AMP-dependent synthetase and ligase; the gene locus_tag TRIATDRAFT_39887 encodes for a NRPS related to NPS6 (siderophore) of *Cochliobolus heterostrophus*; the gene locus_tag TRIATDRAFT_140190 encodes for a hypothetical protein. Gene clustering is represented by the arrows superposed on the horizontal black line. Intergenic spaces are not drawn in scale.

With respect to genes involved in the formation of siderophores, our antiSMASH-6.0-based analysis demonstrated that in *T. hamatum* FBL 587 a NRPS gene cluster (node_221; 29,375 nt; Supplementary Table S6) showed a composition 100% similar to the known BGC for the production of extracellular siderophore dimethylcoprogen (MIBiG accession BGC0001249) from *Alternaria alternata* [40,41]. The putative dimethylcoprogen gene cluster in the genome of *T. hamatum* FBL 587 consisted of at least of eight genes (Figure 3), which encode for a homologue to transmembrane transporter (ProtID g7985), a homologue to ABC transmembrane transporter (ProtID g7986), a homologue to oxidoreductase (ProtID g7987), a homologue to the siderophore iron transporter (ProtID g7988), a homologue to acetyltransferase activity (ProtID g7989), a homologue to long-chain acyl-CoA synthetase (ProtID g7990), a NRPS protein (ProtID g7991), and a hypothetical protein (protein of unknown function; ProtID g7992). The putative extracellular siderophore dimethylcoprogen gene cluster was also identified in the genome of *T. hamatum* GD12, *T. atroviride* IMI 206040, *T. gamsii* T6085, *T. asperellum* CBS 433.97, and *T. lixii* MUT3171 (Supplementary Tables S7–S11).

**Figure 4:**
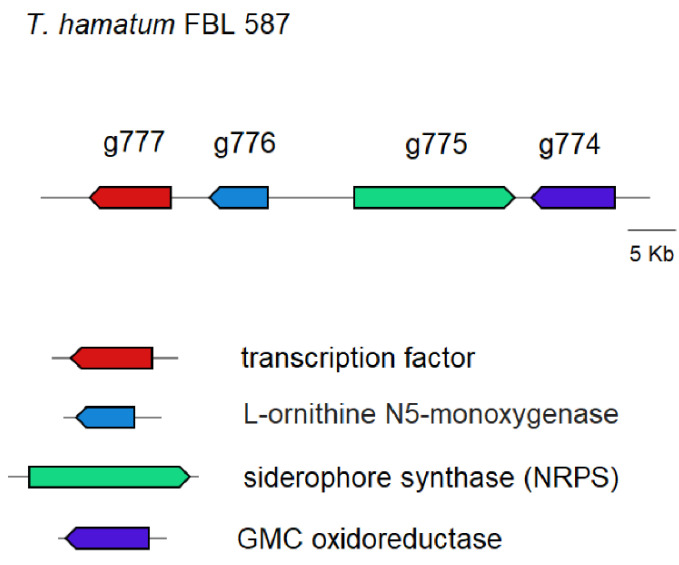
The putative ferricrocin siderophore gene cluster in the genome of *T. hamatum* FBL 587 consisted of four genes, which encode for an GMC oxidoreductase (ProtID g774), a (NRPS) ferricrocin synthase (ProtID g775), a L-Ornithine-N5-oxygenase (ProtID g776), and a transcription factor (ProtID g777). Gene clustering is represented by the arrows superposed on the horizontal black line. Intergenic spaces are not drawn in scale.

In the genome of *T. hamatum* FBL 587 we also identified a hypothetical gene cluster with a NRPS siderophore synthase (ProtID g775) encoding gene orthologue to that found in other *Trichoderma* species for the production of the intracellular siderophore ferricrocin [42]. An analysis of the *T. hamatum* FBL 587 genome revealed the presence of ferricrocin synthesis related genes comprised in the cluster, namely a transcription factor (ProtID g777), L-Ornithine-N5-oxygenase (ProtID g776), and an GMC oxidoreductase (ProtID g774), as shown in the Figure 4.

### 3.4. Prediction of Genes Involved in the Metabolism of DDT

By genomic analysis of *T. hamatum* FBL 587, we investigated the occurrence of genes potentially associated to DDT tolerance. Especially, protein function prediction for enzymes through GO terms allowed to specify molecular functions and potentially involvement in metabolism of aromatic compounds such as DDT. In the FBL 587 genome, there were candidate genes that might contribute to the upstream metabolism of aromatic compounds including ligninolytic enzymes such as a multicopper oxidase or extracellular laccase (ProtID g9164). We also identified genes encoding intracellular non-ligninolytic enzymes that can potentially mediate the initial (upstream) oxidation of aromatic ring structures. The degradation pathway of DDT may involve genes encoding metabolizing enzymes such as cytochrome P450 monooxygenases (CYP450s), of which the presence was revealed from the FBL 587 genome. The complete cytochrome P450 complement (CYPome) involved in resistance to polyaromatic hydrocarbons and xenobiotic detoxification (the so-called phase I enzymes; [9]) has been established from sequenced species of *Trichoderma* [43]. Our genomic analysis demonstrated the presence of several genes encoding cytochrome P450 monooxygenases, such as ProtID g10800, g39, g9970, g6724, g1439, g7450, g128, g8100, g9579, g3317, and g3944. In particular, *T. hamatum* FBL 587 has three genes encoding monooxygenases that contain domains indicating their specificity to benzoate (ProtID g39, g7471, and g9970), and one monooxygenase has specificity to n-alkane (ProtID g4242). 

Additional genes that are potentially involved in the downstream steps for DDT transformation were also investigated. We identified putative genes encoding epoxide hydrolases (e.g., ProtID g352, g1543), some of which might have functions in detoxification [44]. Candidate genes encoding oxidoreductase enzymes including alcohol dehydrogenases (e.g., ProtID g7969), aldehyde dehydrogenases (e.g., ProtID g5709, g6345 and g8931), NAD-dependent aldehyde dehydrogenases (ProtID g5178, g6146, and g773), aldehyde dehydrogenases NAD (ProtID g8129, and g6040), one short-chain dehydrogenase (ProtID g1254), and FAD-dependent monooxygenases (e.g., ProtID g33, g9198, g4149, g10870, g5490, g985, g5871, and g9407) were found in the genome of FBL 587. Genes encoding dioxygenases comprising one putative hydroxyquinol-dioxygenase (ProtID g10302), and one aromatic compound dioxygenase (ProtID g10767) were also found in the *T. hamatum* FBL 587 genome.

Additionally, degradation of compounds such as DDT or PAHs relies on transferases (the so-called phase II conjugating enzymes; [9]) that make such polyaromatic compounds more hydrosoluble and less toxic. Our results confirmed that the *T. hamatum* FBL 587 genome possesses genes encoding for glutathione S-transferases (GSTs), glycosyltransferases (GTFs), and sulfotransferases, which can catalyse the addition of glutathione, glycosyl, or sulphate donors to DDT, respectively. Specifically, 6 genes encoding putative GSTs (ProtID g920, g5585, g1796, g8350, g9635, and g8655), one GTF (ProtID g3303) and one sulfotransferase (ProtID g2564) were encoded for the potential conjugation of DDT.

According to GO functional analysis, the genes encoding transmembrane transporters (the so-called phase III coding for transporters; [9]) were also identified. These included several MFS general substrate transporters (e.g., ProtID g1350, g3396, g10401, g3264, g8078, g3803, g10322, g9334, g10400, g2310, g1362, g4159, g4828, g1309, g9714, g3742, g700, g1256, g2260, g5719, g845, g3533, and g8876), and ABC transporters (e.g., ProtID g6498, g6886, g7790, g8027, and g3411).

Finally, candidate genes encoding antioxidant enzymes, such as GSTs (see above), superoxide dismutase (SOD; ProtID g4389, and g6464) and catalase (ProtID g5890) were also found. 

### 3.5. Genome-Wide Transcriptomic Responses of T. hamatum FBL 587 during DDT Exposure 

**Figure 5:**
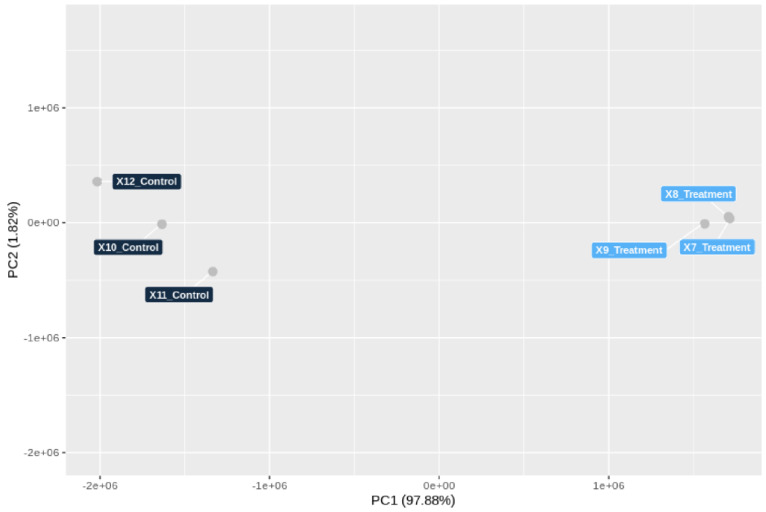
Principal Component Analysis (PCA) conducted on the normalized gene expression values of the samples (control and DDT treatment). X- and Y-axes show the PC1 and PC2, respectively, with the amount of variance explained by each component reported in parenthesis. Each point in the plot represents a sample, dots of the same colours are replicates of a same experimental group. PCA showed that replicates cluster together for both control and treatment, and the treatment group is clearly distinct from the control group.

In order to investigate which of the genes in *T. hamatum* FBL 587 were upregulated during DDT exposure in FBL 587, a genome-wide transcriptomic analysis was performed. Specifically, *T. hamatum* FBL 587 grown for 48 h with DDT (10 mg/L) was subjected to RNA-Seq analysis. For each library of RNA-Seq data, over 21–24 million reads were mapped back in pairs for both biological replicates of treated and untreated samples (Supplementary Table S1). After the alignment of the reads against the FBL 587 genome, PCA of the normalized gene expression values as input clearly separated DDT-added group (3 samples) from the control group (Figure 5). The PCA clustered DDT samples together, which formed an independent group distinct from the untreated samples (control), indicating that the gene expression profile of the DDT treatment was different from the control.

**Figure 6:**
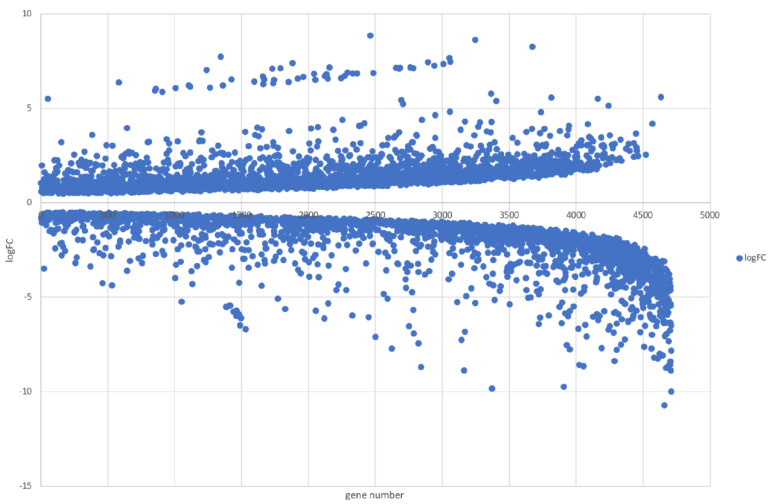
A plot reporting results from transcriptomic analysis for *T. hamatum* FBL 587 following exposure to DDT (10 mg/L for 48 h), in which blue dots represent the genes (4710) that were significantly expressed (FDR ≤ 0.05) by exposure to DDT, with log2FC ≥ 1 for upregulated genes (1706) and log2FC ≤ −1 for downregulated genes (1770), where x-axis is the gene number and y-axis is log2FC value. Abbreviations: FDR, False Discovery Rate (Benjamini-Hochberg correction); log2FC, fold change relative to control.

Of the 10,994 genes predicted in the FBL 587 genome, the RNA-Seq read counts to each annotated locus identified 4710 genes whose expression was regulated by DDT (FDR value ≤ 0.05), as they are reported in Figure 6. Among the significantly differentially expressed genes following exposure to DDT (FDR ≤ 0.05), those with log2FC (fold change relative to control) ≥ 1 were upregulated genes (1706) and those with log2FC ≤ −1 were downregulated genes (1770). However, the molecular function of some genes regulated by DDT could not be predicted or assigned to GO classes.

### 3.6. Transcriptomic Analysis of DDT Tolerance Genes by T. hamatum FBL 587

In *T. hamatum* FBL 587, xenobiotic detoxification genes i.e., phase I coding for cytochrome P450 monooxygenases (CYP450s), phase II conjugating enzymes, and phase III coding for ABC efflux and MFS transporters [9] were transcriptionally upregulated (FDR ≤ 0.05, log2FC ≥ 1), resulting in its resistance to DDT. 

With regard to phase I, the CYP450 genes ProtID g128 (cytochrome P450 monooxygenase sdnT) and ProtID g8100 (cytochrome P450 monooxygenase), which showed high expression (log2FC ≥ 1) when *T. hamatum* FBL 587 was cultured on medium with DDT, would contribute to *T. hamatum* tolerance to DDT. 

Among the phase II conjugating enzymes, the genes encoding transferases such as GTFs (ProtID g3303) and GSTs (ProtID g1796 and g8655), which make polyaromatic compounds such as DDT or PAHs more hydrosoluble and less toxic, were significantly induced (FDR ≤ 0.05, log2FC ≥ 1) following exposure to DDT.

With regards to phase III, our genomic and transcriptomic analysis showed that in the comparison DDT treatment vs control, the genes encoding transmembrane transporters were significantly differentially expressed. Particularly, upregulation by DDT was evidenced (FDR ≤ 0.05, log2FC ≥ 1) in 23 MFS transporters (ProtID g1350, g3396, g10401, g3264, g8078, g3803, g10322, g9334, g10400, g2310, g1362, g4159, g4828, g1309, g9714, g3742, g700, g1256, g2260, g5719, g845, g3533, and g8876), suggesting an important role of these transporters for cellular response to DDT. According to GO functional enrichment analysis, we also identified ABC transporters that were upregulated (e.g., ProtID g8027; FDR ≤ 0.05, log2FC > 1) during DDT exposure, suggesting their putative involvement in the translocation of DDT. 

The DDT-specific upregulation of genes potentially involved in enzymatic antioxidant defence mechanisms such as superoxide dismutase (SOD, ProtID g6464; FDR ≤ 0.05, log2FC > 1) and catalase (ProtID g5890; FDR ≤ 0.05, log2FC > 1) was also observed, which in *T. hamatum* FBL 587 can be involved in the resistance to DDT. 

### 3.7. Transcriptomic Analysis of DDT Transforming Genes by T. hamatum FBL 587

In addition to the genes involved in the detoxification of DDT by transport and metabolism (phase I, II, III; [9]), the DDT-upregulated 1706 genes included putative DDT-transforming genes. We investigated the expression profiles of enzymes for initial aromatic-ring oxidation, i.e., extracellular laccase and intracellular P450s. In DDT-treated samples of *T. hamatum* FBL 587, laccase (ProtID 9164) was not upregulated (FDR value > 0.05). However, for this gene the treatment replicates showed a higher level of expressed transcripts than control replicates. With regards to intracellular P450s, some of them were upregulated, as mentioned above.

In *T. hamatum* FBL 587, DDT-responsive genes potentially responsible for the downstream steps for DDT transformation were also identified. The expression patterns of these genes showed that most of them were upregulated during DDT exposure. The genes encoding epoxide hydrolases have potential functions in detoxification of xenobiotics, reducing the toxic effects of these molecules [44]). Particularly, in *T. hamatum* FBL 587 under DDT exposure the epoxide hydrolase (ProtID g352) resulted in an upregulated gene (FDR ≤ 0.05, log2FC > 1) in the process of detoxification. Moreover, a putative secreted hydrolase (ProtID g1645) resulted in significantly upregulation (log2FC = 6.5).

Among the DDT-responsive genes, a Dye-decolorizing peroxidase (DyP-type peroxidase, ProtID g3541), some alcohol dehydrogenases (ProtID g7969), including zinc-containing alcohol dehydrogenase (ProtID g8841, g9598), and NADP-dependent alcohol dehydrogenase (ProtID g7677, and g1942) were significantly upregulated (FDR ≤ 0.05, log2FC > 1). Moreover, aldehyde dehydrogenases (ProtID g5709, and g6345), NAD-dependent aldehyde dehydrogenase (ProtID g5178, g6146), aldehyde dehydrogenase NAD (ProtID g8129, g6040) were upregulated (FDR ≤ 0.05, log2FC > 1). We also observed that two FAD-dependent monooxygenases (ProtID g9407, and g9198), and two GSTs (ProtID g1796, and g8655), and one GTF (ProtID g3303) were upregulated (FDR ≤ 0.05, log2FC ≥ 1). Interestingly, the *g22* gene encoding salicylate hydroxylase, which catalyses essential reactions at the junction between the so-called upper and lower catabolic pathways, presented high transcript levels (FDR ≤ 0.05, log2FC > 1) when *T. hamatum* FBL 587 was exposed to DDT, indicating its importance in the response to DDT.

Finally, via the genomic analysis of *T. hamatum* FBL 587, the genetic potential of *T. hamatum* FBL 587 to produce biosurfactants was investigated. Among these genes, our NGS RNA-Seq data indicated that the genes encoding hydrophobins cerato-platanins (ProtID g2774, g5696, and g150) and cerato-ulmins (ProtID 2606) were not upregulated during the two days of DDT exposure. However, the 1706 DDT-upregulated genes included genes involved in lipid transport and metabolism, in which lipases (ProtID g8227 and g4462) were upregulated (FDR ≤ 0.05, log2FC > 1). *Trichoderma hamatum* FBL 587 contains key genes for trehalose synthesis with the potential to extracellular trehalose lipid biosurfactants, namely trehalose-phosphate synthase (ProtID g2142), and trehalose-phosphate phosphatase (ProtID g9403). Although the trehalose-phosphate synthase was not upregulated, it resulted a differentially expressed gene (log2FC = 0.7) during the two days of exposure to DDT.

## 4. Discussion

### 4.1. Genome Properties of T. hamatum FBL 587

*Trichoderma hamatum* FBL 587 showed a remarkable DDT-resistance, as reported in Russo et al., [5]. Moreover, results from this study suggest a putative ability of *T. hamatum* FBL 587 to enhance DDT degradation process in soil. Here we present the 38.9 Mb-genome sequence of *T. hamatum* FBL 587, 98.5% complete as predicted by BUSCO, and transcriptomic analysis to observe differential expression of genes by DDT. For the first time, in *T. hamatum* FBL 587 we provide molecular evidence showing the DDT induced over-expression of several genes correlated with tolerance to DDT and putatively with DDT metabolism. 

The genome size of *T. hamatum* FBL 587 is essentially similar to that of other sequenced *T. hamatum* strains, namely YYH13 (38.93 Mb; [13]), YYH16 (38.92 Mb; [13]), and GD12 (38.43 Mb; [14,15]). Moreover, the annotated 10,994 protein-coding genes in FBL 587 fell within the known range for the genomes of the *T. hamatum* strains YYH13 (11,316), YYH16 (11,755) and GD12 (11,203), as reported in [13]. The 1.2% repetitive elements found in the genome of *T. hamatum* FBL 587 was typical among the sequenced *T. hamatum* strains, i.e., 1.47% repetitive elements for YYH13, 1.58% for YYH16, and 1.31% for GD12 [13]. Therefore, these NGS data indicate similar genome properties, reflecting a recent common ancestor. 

This finding is also supported by further genomic analysis. In *T. hamatum* FBL 587 using the dbCAN CAZyme database we revealed the presence of a range of genes encoding for CAZymes that can be comparable with that of previously sequenced *T. hamatum* strains YYH13, YYH16 and GD12 [13,14,15], reflecting the adaption to their ecological niches as saprotrophic *Trichoderma* species in soil and rhizosphere, competing with other soil microorganisms. With respect to the GH class of CAZymes, the number of members of the glycoside hydrolases found in FBL 587 was similar to that harboured by other sequenced strains (YYH13, YYH16, and GD12) of *T. hamatum*. For instance, the cellulolytic CAZyme composition in *T. hamatum* strains YYH13, YYH16 and GD12 [13,14,15] was represented by the presence of frequent chitinases (GH18). Consistently with the findings, the genome of *T. hamatum* FBL 587 revealed 28 copies of GH18 and 3 copies of GH20. Moreover, among the carbohydrate hydrolases, the number of cellulases (e.g., GH6 = 1, GH7 = 2), glucanases (GH55 = 8, GH12 = 3, GH17 = 2, GH81 = 2 and GH64 = 3), xylanases (e.g., GH10 = 3, GH11 = 4), β-glucosidases and β-galactosidases (GH1 = 3), α-glucosidases (GH31 = 7), β-galactosidases (GH35 = 2) and α-mannanases (GH76 = 8) found in *T. hamatum* FBL 587 was significantly similar to that of other previously sequenced strains of *T. hamatum* [13].

The SM BSGC potential of *T. hamatum* FBL 587, which includes PKSs, NRPSs and terpenes, was compared with those of other *Trichoderma* species of the section *Trichoderma* (see [15]), such as *T. atroviride* IMI 2060, which is publicly available at the antiSMASH database [30], and *T. lixii* MUT3171 [4] of the clade *Harzianum/Virens* (see [15]) as outgroup. Most of the antiSMASH-6.0-detected gene clusters cannot be annotated in *T. hamatum* FBL 587 as their composition is not similar to any known fungal BGC. Apparently, putative BSGCs for mycotoxin production were not detected in *T. hamatum* FBL 587, which indicates that mycotoxin production does not play a major role in this strain, thus making the strain an ideal, harmless, candidate to avoid the risk of mycotoxin contamination during bioremediation. However, the SM responses of *T. hamatum* FBL 587 in the presence of DDT as well as the association between the ability to biodegrade DDT and other pollutants and mycotoxin production are to be experimentally verified, possibly under conditions closer to the level of soil DDT contamination [2], considering also factors such as light, pH, nutrients that can be relevant in the fungal mycotoxin production [42].

*Trichoderma* species are reported to synthesize a broad diversity of terpenoids by terpene synthases [45], including MT γ-terpinene and the sesquiterpene (SQT) γ-candinene that were the most abundant VOCs of *T. hamatum* [46]. In *T. hamatum* FBL 587, terpenes have not been experimentally characterized yet, but in its genome we have identified terpene synthase genes (e.g., *g7460*, *g9669*, and *g3784*) potentially involved in terpene biosynthesis [47]. Most of them apparently were not upregulated after two days of exposure to DDT. However, in *T. hamatum* FBL 587 a BGC putatively involved in the biosynthesis of squalestatin S1 or squalestatin analogues [37], homologue to the BGC found in other *Trichoderma* species including *T. atroviride* IMI 2060 (antiSMASH database; [29,30]), contained a squalene synthase (ProtID g4482) that was upregulated under two days of exposure to DDT, but a deeper analysis is needed to draw informed conclusions. Moreover, our antiSMASH-based analysis found a putative T1PKS gene cluster for alternariol biosynthesis in the genome of *T. hamatum* FBL 587. Alternariol, a compound with antioxidant activity [36], was obtained from the fermentation broth of *Trichoderma* sp. Jing-8 [48,49]. However, our RNASeq data showed that the T1PKS gene (*g8761*) belonging to this putative gene cluster was not upregulated by DDT exposure, which indicates alternariol biosynthesis is not involved in oxidative stress tolerance. Moreover, we did not observe upregulation by DDT exposure in *T. hamatum* FBL 587 for the gene *g1187* encoding a terpene cyclase/mutase belonging to the putative biosynthetic gene cluster for clavaric acid. A detailed comparative analysis including other *Trichoderma* species to demonstrate genomic organization of terpene BGCs is worthy of further investigations, but it goes beyond the scope of the present paper.

Interestingly, a NRPS gene (g7991) cluster in *T. hamatum* FBL 587 showed an identical composition to the known BGC for the extracellular siderophore dimethylcoprogen [40,41] and was upregulated in the presence of DDT. Another NRPS gene (*g775*) homologue of the NRPS gene responsible for the synthesis of ferricrocin [50] was identified in *T. hamatum* FBL 587 genome, which was arranged in a distinct cluster. However, even though it is known that the siderophore ferricrocin is used for intracellular storage of iron and that it is involved in the protection of fungal cells from oxidative stress [51], the *g775* was not upregulated by exposure to DDT. Therefore, although the role of siderophores in fungal resistance to oxidative stress was proposed in previous studies [4,40,41], their contribution to the resistance against DDT-induced ROS should be evaluated under varying DDT contamination and exposure conditions. 

### 4.2. Transcriptomic Analysis of DDT Tolerance Genes by T. hamatum FBL 587

According to our analyses, *T. hamatum* FBL 587 under DDT exposure upregulated phase I, II, III genes encoding different enzymes potentially involved in modification and translocation of DDT. Considering the results at the transcription of the phase I genes that regards genes encoding for CYP450s, our analysis revealed that DDT induced overexpression of genes encoding for cytochrome P450 monooxygenase sdnT (ProtID g128) and cytochrome P450 monooxygenase (ProtID g8100). Cytochrome P450 monooxygenases of fungi are involved in many essential cellular processes and play diverse roles since they catalyse the conversion of hydrophobic intermediates and have been found to support the growth of fungi also in the presence of environmental pollutants [52].

With regard to phase II, our analysis also revealed genes encoding transferases in *T. hamatum* FBL 587. In the comparison DDT treatment vs control, the gene encoding transferases such as GTFs (ProtID g3303) and GSTs (ProtID g1796 and g8655), which make polyaromatic compounds such as DDT more hydrosoluble and less toxic, were upregulated in response to DDT. For instance, we hypothesized that in *T. hamatum* FBL 587, glycosyltransferases may participate to phase II reactions in the DDT modification pathway: after the initial attack by oxidoreductases, these enzymes may transfer an activated sugar residue to the hydroxyl groups of the DDT metabolites, which may be secreted. 

DDT exposure induced in *T. hamatum* FBL 587 the transcription of a significant number of genes, with a distinct transcriptomic response explained by genes putatively involved in translocation of DDT out of cells (phase III). For instance, some ABC transporters (e.g., ProtID g8027) were upregulated in response to DDT. This observation is consistent with previous studies in *Trichoderma* in which ABC transporters contributed to resistance against toxic compounds and were involved in protection from exogenous toxicants [9]. Also, in *T. hamatum* FBL 587 we found the transcription of a significantly number of genes encoding the transmembrane transporters MFS, which are known for their ability to actively export xenobiotic and/or metabolized compounds across the cell membranes. On the basis of these results, we hypothesized putative translocation of DDT by means of MFS transporters that not only could contribute to resistance against DDT but also could enable modification of DDT.

The transcription factor that probably is responsible for induced overexpression of the phase I, II and III genes would be worthy of further investigations. We hypothesized that it might be regulated by a putative xenobiotic detoxification regulator such as one of the members of the fungus-specific Zn2Cys6 family, which have been known to be the regulator of fungal ABC transporters [9]. 

Particularly worthy of note is the fact that the comparison between cells growth under DDT exposure vs control evidenced among the upregulation of genes encoding oxidoreductases of *T. hamatum* FBL 587, a significantly differential expression of a gene for superoxide dismutase (ProtID g6464). It is well known that within the soil community, *Trichoderma* species respond to ROS originated during the plant-pathogenic fungi interactions by enhancing the expression of genes that encode for oxidative stress response and detoxification enzymes [12]. Since DDT is known to increase the production of ROS in *T. hamatum* FBL 587, leading to detectable oxidative stress [5], significant upregulation of ROS scavenging enzymes in cells exposed to DDT was expected and was confirmed by the transcriptomic analysis. 

### 4.3. Transcriptomic Analysis of DDT Transforming Genes by T. hamatum FBL 587

DDT biodegradation in soil was assumed to be mediated by *T. hamatum* FBL 587, since its inoculation favoured the biodegradation of the compound into DDT isomers and its metabolites DDE and DDD and decreased the overall DDT concentration in soil by inducing an increased accumulation of the pollutants in roots of *C. pepo*. It is worth noting that all the genes involved in the fungal DDT biodegradation pathway have not yet been elucidated. Therefore, the putative DDT metabolism by *T. hamatum* FBL 587 requires to be corroborated by degradation studies with DDT as sole carbon source. Moreover, the effect of autochthonous bacterial enzymes such as DDT-dehydrohalogenases that can be involved in the catalytic degradation of DDT to form DDD/DDE [53] cannot be ruled out in our DDT-contaminated soil. 

Although the genetic details of fungal DDT degradation have not been established, the genomic and transcriptomic analysis of *T. hamatum* FBL 587 carried out in this study revealed a total of 1706 genes upregulated by DDT, many of which having known molecular functions. Several of these genes were encoding for enzyme systems potentially involved in xenobiotic degradation such as multicopper laccases and peroxidases. The presence of gene products in the RNA-Seq analysis of *T. hamatum* FBL 587 identified as an extracellular laccase could also suggest the putative involvement of this enzyme in the biodegradation process, even though it was not upregulated during the two days of DDT exposure. Indeed, the degradation of PAHs by laccase activity of *T. asperellum* was nearly undetectable during the initial two days but increased significantly after four days [7]. 

The initial steps of PAH metabolism in many filamentous fungi involve the action of cytochrome P450 monooxygenases and epoxide hydrolases [52]. The analyses performed in this study suggested that the ability of *T. hamatum* FBL 587 to metabolize DDT lies in key enzymatic reactions of aerobic biodegradation catalysed by peroxidases and cytochrome P450 oxidases. Indeed, in response to DDT, it upregulated genes for cellular oxidant detoxification, such as a catalase-peroxidase (ProtID g5890), a putative secreted hydrolase (ProtID g1645), and a DyP-type peroxidase (ProtID g3541). Such gene regulation pattern would suggest that in *T. hamatum* FBL 587 the cleavage of carbon-chlorine bonds in organochlorine compounds degradation may occur via enzymatic dechlorination catalysed by enzymes such as monooxygenases.

Moreover, *T. hamatum* FBL 587 also upregulated genes encoding intracellular enzymes such as cytochrome P450 monooxygenase (CYPs or P450s), which are key enzymes in fungal xenobiotic detoxification [54]. P450 enzymes have been reported to metabolize DDT under aerobic conditions as well as to be involved in the degradation of some polyaromatic pollutants such as PAHs [4]. In *Trichoderma* species, cytochrome P450s form an important group of enzymes involved in degradation of environmental pollutants [43]. It can be assumed that intracellular P450 enzymes also have an important role in the metabolism of persistent aromatic compounds in *T. hamatum* FBL 587. Its transcriptomic analysis revealed that P450s might be responsible for the initial oxidation of aromatic rings. A previous genome-scale identification of P450 genes in *T. lixii* showed potential DDT-oxidizing activity of these enzymes [4]. Understanding the molecular mechanisms of fungal P450 genes involved in DDT metabolism of *T. hamatum* FBL 587 is the objective of ongoing study.

Intriguingly, the gene *g4985* encoding benzoate 4-monooxygenase, which is responsible for cytochrome P450 dependent benzoate hydroxylation in microsomes [55], was upregulated, while three genes for putative benzoate 4-monooxygenase cytochrome P450 (ProtID g39, g7471, and g9970) were not. Moreover, DDT exposure induced upregulation and differentially expression of two genes encoding dihydroxybenzoate decarboxylase (ProtID g24, g7819). This enzyme is involved in the pathway benzoate degradation via hydroxylation, which is part of aromatic compound metabolism [56]. On the other hand, the gene *g21* for catechol 1,2-dioxygenase showed higher expression when *T. hamatum* FBL 587 was cultured on medium with DDT than control, but apparently it was not significantly upregulated during the two days of exposure to DDT. It thus appeared that the regulation of related genes could be differentially modulated by the xenobiotic stress [57].

In addition to P450s, in response to DDT we observed upregulation of many other genes encoding potential DDT-transforming enzymes such as alcohol dehydrogenase (ProtID g8841, g9598), aldehyde dehydrogenase (e.g., ProtID g8129), FAD-dependent monooxygenases (ProtID g9407 and g9198), GTF (ProtID 3303), and GST (ProtID 1796, 8655). Particularly worthy of note is the fact that at least eight aldehyde dehydrogenases were identified in the *T. hamatum* FBL 587 genome and five of them were upregulated in terms of mRNA levels after cell exposure to DDT. Aldehyde dehydrogenases could exhibit oxidase activities to catalyse the oxidation of DDT intermediate, such as phenoxybenzyl aldehyde to phenoxybenzoic acid [58]. Moreover, the gene *g22* for salicylate hydroxylase was also upregulated in response to DDT exposure, which in the microbial degradation of polycyclic aromatic hydrocarbons, catalyses essential reactions at the junction between the so-called upper and lower catabolic pathways [59].

Upregulation of *T. hamatum* FBL 587 genes that might increase the production of lipid biosurfactants to emulsify DDT and of genes involved in lipid transport and metabolism, among them lipases (ProtID g8227, g4462), in response to exposure to DDT could also be associated to the role that biosurfactants with bioemulsifier activities may have in biotransformation of aromatic compounds though enhancing their hydrosolubility and bioavailability into the soil [4]. *Trichoderma hamatum* FBL 587 resulted to have also the genetic potential to produce other biosurfactants such as hydrophobins, which genes were deduced from genomic sequences of *Trichoderma* species [12]. In saprophytic *Trichoderma* species established in soil and rhizosphere, hydrophobin-like proteins are involved in attachment of *Trichoderma* to roots [42]. In agreement with this finding, in the genome of *T. hamatum* FBL 587 genes encoding hydrophobin cerato-platanins and cerato-ulmins (small amphiphilic proteins) were found, but NGS RNA-Seq data showed that they were not upregulated during two days of DDT exposure. However, in *T. hamatum* FBL 587 the gene encoding trehalose-phosphate synthase (ProtID g2142), a key gene for trehalose synthesis, resulted to be transcripted, though at low level, in response to the 2-day DDT exposure stress, suggesting the involvement, to some extent, of the trehalose metabolism in increasing the tolerance to DDT. It seems reasonable to assume that the production of extracellular trehalose lipid biosurfactants with emulsifying properties would contribute to increase tolerance of *T. hamatum* FBL 587 to DDT and/or to the enhanced uptake of DDT from the plant root system, as observed in the pot trial.

## 5. Conclusions

The whole-genomic and transcriptomic analysis of *T. hamatum* FBL 587, performed for the first time, provided insights into the complex gene responses of this strain when challenged by DDT exposure. Several *T. hamatum* FBL 587 genes were upregulated in association with DDT exposure, which suggest their putative roles in DDT metabolism. However, the elucidation of the exact molecular mechanisms associated with them requires further studies. In addition, upregulation of many genes with unknown molecular functions provides a clue for future research into the identification of novel genes involved in DDT biodegradation. Further studies involving RNA-Seq data are necessary to characterize the transcriptional regulation of the system of DDT detoxification genes in *T. hamatum* FBL 587. Understanding the genetic aspects involved in DDT metabolization could support the exploitation of *T. hamatum* FBL 587 for the mycoremediation of DDT-polluted environments. Future research should include molecular approaches that allow real-time comparison of the functionality of different genes inducing tolerance or degradation of DDT for the efficient screening and application of this strain in mycoremediation strategies. 

## Figures and Tables

**Table 1:** Summary of the *T. hamatum* FBL 587 genome sequencing and assembly results.

Parameter	Estimated Value
Number reads	6,329,382
Number used reads	6,251,876
Number of contigs (≥500 bp)	1354
Largest contig	347,610
N50 reads	69,288
L50 contigs	166
Predicted genes	10,944
Genome size	38,965,975 bp
GC content	48.54%
tRNA	230
8S rRNA	50
18S rRNA	1
28S rRNA	1
Repeat class/family	
Simple repeats	399,867 (1%)
Low complexity	79,381 (0.2%)
Completeness	98.5%
Fragmented	0.7%
Missing	0.2%
Complete and duplicated copy	0.6%

**Table 2:** The identification and characterization of PKS, NRPS, PKS-NRPS hybrids and terpene gene clusters in *T. hamatum* FBL587 and other four *Trichoderma* species of the section *Trichoderma* (see [15]) performed with antiSMASH v. 6.0.0 (see [29]). The genome of *T. lixii* MUT3171 [4] of the clade *Harzianum*/*Virens* (see [15]) is included in the antiSMASH-6.0 analysis, as it was chosen to represent the outgroup species. All *Trichoderma* genome sequences were taken from NCBI databases and have been published [4,14,15,34,35]. Genome assemblies are available at DDBJ/EMBL/GenBank under the following GenBank accessions: *T. atroviride* IMI 206040, ABDG02000000; *T. gamsii* T6085, JPDN00000000; *T. asperellum* CBS 433.97, MBGH00000000; *T. hamatum* GD12, ANCB00000000; *T. lixii* MUT3171, SESN00000000.

Genome	NRPS, T1PKS	NRPS	NRPS-Like	T1PKS	Terpene	Total
*Trichoderma asperellum* CBS 433.97	6	10	10	11	11	48
*Trichoderma atroviride* IMI 206040	4	10	10	12	8	44
*Trichoderma gamsii* T6085	4	9	11	12	7	43
*Trichoderma hamatum* GD12	5	5	6	7	5	28
*Trichoderma hamatum* FBL 587	4	4	5	8	6	27
*Trichoderma lixii* MUT3171	9	4	7	19	8	47

## Data Availability

The whole genome sequence project of FBL 587 has been deposited at DDBJ/ENA/GenBank under the accession SEIV00000000. The RNA-sequencing data have been deposited in NCBI SRA under the IDs: SRR14056734-9.

## Supplementary Materials

The following are available online at https://www.mdpi.com/article/10.3390/microorganisms9081680/s1, Figure S1: Low expression filtering step on transcriptome sequencing data from *Trichoderma hamatum* FBL 587; the algorithm calculated a Global Jaccard index of similarity (on the Y-axis) between the samples in function of different minimum normalized read counts (on the X-axis). The graphic shows the threshold at s = 13.294 in which the replicates have the highest similarity; Table S1: Number of NGS-RNASeq reads before and after quality check on the raw sequencing data from *Trichoderma hamatum* FBL 587; Table S2: Trimmed Mean of M-values (TMM) obtained by normalization applied to the raw fragment from *Trichoderma hamatum* FBL 587; Table S3: Reads Per Kilobase Million (RPKM) obtained by normalization applied to the raw fragment from *Trichoderma hamatum* FBL 587; Table S4: The concentrations of DDT metabolites and isomers detected in soil and plant organs (mg/kg) of two *Cucurbita pepo* accessions inoculated with *Trichoderma hamatum* FBL 587. ND—not detected, ΣDDT = sum of DDT metabolites and isomers; Table S5: Carbohydrate active enzymes (CAZymes) in *Trichoderma hamatum* FBL 587 identified with the dbCAN2, which integrates three different tools: (1) HMMER, (2) DIAMOND, (3) Hotpep (see text); Table S6: The secondary metabolite gene clusters identification in *Trichoderma hamatum* FBL 587 performed with antiSMASH v. 6.0.0 (see text); Table S7: The secondary metabolite gene clusters identification in *Trichoderma asperellum* CBS 433.97 performed with antiSMASH v. 6.0.0 (see text); Table S8: The secondary metabolite gene clusters identification in *Trichoderma atroviride* IMI 206040 performed with antiSMASH v. 6.0.0 (see text); Table S9: The secondary metabolite gene clusters identification in *Trichoderma gamsii* T6085 performed with antiSMASH v. 6.0.0 (see text); Table S10: The secondary metabolite gene clusters identification in *Trichoderma hamatum* GD12 performed with antiSMASH v. 6.0.0 (see text); Table S11: The secondary metabolite gene clusters identification in *Trichoderma lixii* MUT3171 performed with antiSMASH v. 6.0.0 (see text).
