# Supplementary material for: A Genomic and Transcriptomic Study on the DDT-Resistant Trichoderma hamatum FBL 587: First Genetic Data into Mycoremediation Strategies for DDT-Polluted Sites
Source: Microorganisms. 2021 Aug 7;9(8):1680. doi: 10.3390/microorganisms9081680 (PMC8401308; doi:10.3390/microorganisms9081680)
Supplement: Supplementary file 1 [file microorganisms-09-01680-s001.zip › Supplementary Table Figure caption.pdf]

Supplementary Table S1. Number of NGS-RNASeq reads before and after quality check on the raw sequencing data from *Trichoderma hamatum* FBL 587.

Supplementary Table S2. Trimmed Mean of M-values (TMM) obtained by normalization applied to the raw fragment from *Trichoderma hamatum* FBL 587.

Supplementary Table S3. Reads Per Kilobase Million (RPKM) obtained by normalization applied to the raw fragment from *Trichoderma hamatum* FBL 587.

Supplementary Table S4. The concentrations of DDT metabolites and isomers detected in soil and plant organs (mg/kg) of two *Cucurbita pepo* accessions inoculated with *Trichoderma hamatum* FBL 587.
ND – not detected, ΣDDT = sum of DDT metabolites and isomers.

Supplementary Table S5. Carbohydrate active enzymes (CAZymes) in *Trichoderma hamatum* FBL 587 identified with the dbCAN2, which integrates three different tools: (1) HMMER, (2) DIAMOND, (3) Hotpep (see text).

Supplementary Table S6. The secondary metabolite gene clusters identification in *Trichoderma hamatum* FBL 587 performed with antiSMASH v. 6.0.0 (see text).

Supplementary Table S7. The secondary metabolite gene clusters identification in *Trichoderma asperellum* CBS 433.97 performed with antiSMASH v. 6.0.0 (see text).

Supplementary Table S8. The secondary metabolite gene clusters identification in *Trichoderma atroviride* IMI 206040 performed with antiSMASH v. 6.0.0 (see text).

Supplementary Table S9. The secondary metabolite gene clusters identification in *Trichoderma gamsii* T6085 performed with antiSMASH v. 6.0.0 (see text).

Supplementary Table S10. The secondary metabolite gene clusters identification in *Trichoderma hamatum* GD12 performed with antiSMASH v. 6.0.0 (see text).

Supplementary Table S11. The secondary metabolite gene clusters identification in *Trichoderma lixii* MUT3171 performed with antiSMASH v. 6.0.0 (see text).

Supplementary Figure S1. Low expression filtering step on transcriptome sequencing data from *Trichoderma hamatum* FBL 587; the algorithm calculated a Global Jaccard index of similarity (on the Y-axis) between the samples in function of different minimum normalized read counts (on the X-axis). The graphic shows the threshold at s = 13.294 in which the replicates have the highest similarity.
