# Supplementary material for: A Genomic and Transcriptomic Study on the DDT-Resistant Trichoderma hamatum FBL 587: First Genetic Data into Mycoremediation Strategies for DDT-Polluted Sites
Source: Microorganisms. 2021 Aug 7;9(8):1680. doi: 10.3390/microorganisms9081680 (PMC8401308; doi:10.3390/microorganisms9081680)
Supplement: Supplementary file 1 [file microorganisms-09-01680-s001.zip › Table S1.pdf]

Supplementary Table S1. Number of NGS-RNASeq reads before and after quality check on the raw sequencing data from *T. hamatum* FBL 587.

Sample name	Raw reads	Trimmed reads
Treatment		
X7	26,243,489	21,106,233
X8	27,183,202	22,544,499
X9	27,559,216	22,606,573
Control		
X10	29,156,404	24,066,207
X11	27,478,579	22,560,427
X12	29,202,771	23,784,059
