# Supplementary material for: A Genomic and Transcriptomic Study on the DDT-Resistant Trichoderma hamatum FBL 587: First Genetic Data into Mycoremediation Strategies for DDT-Polluted Sites
Source: Microorganisms. 2021 Aug 7;9(8):1680. doi: 10.3390/microorganisms9081680 (PMC8401308; doi:10.3390/microorganisms9081680)
Supplement: Supplementary file 1 [file microorganisms-09-01680-s001.zip › Table S4.pdf]

Supplementary Table S4. The concentrations of DDT metabolites and isomers detected in soil and plant organs (mg/kg) of two *C. pepo* accessions inoculated with *T. hamatum* FBL 587**.** ND – not detected, ΣDDT = sum of DDT metabolites and isomers.

Fraction	Accession	Treatment	p,p'-DDD	o,p'-DDD	p,p'-DDE	p,p'-DDT	o,p'-DDT	ΣDDT
**Initial soil**	AMES 26607			0.037	ND	0.011	0.104	ND
	PI 614701			0.037	ND	0.008	0.104	ND
**Soil**	AMES 26607	Control	0.024	ND	0.010	0.075	ND	0.108
	PI 614701	Control	0.031	ND	0.011	0.082	ND	0.124
	AMES 26607	+FBL587	0.026	ND	0.009	0.068	ND	0.104
	PI 614701	+FBL587	0.030	ND	0.012	0.043	ND	0.085
**Roots**	AMES 26607	Control	0.042	0.009	0.017	0.050	0.005	0.123
	PI 614701	Control	0.072	0.016	0.026	0.230	0.021	0.365
	AMES 26607	+FBL587	0.040	0.006	0.021	0.163	0.014	0.243
	PI 614701	+FBL587	0.064	0.015	0.032	0.273	0.029	0.412
**Shoots and leaves**	AMES 26607	Control	0.006	ND	ND	ND	ND	0.006
	PI 614701	Control	0.012	ND	ND	ND	ND	0.012
	AMES 26607	+FBL587	0.013	ND	ND	ND	ND	0.013
	PI 614701	+FBL587	0.012	ND	ND	ND	ND	0.012
**Fruits**	AMES 26607	Control	ND					
	PI 614701	Control	ND					
	AMES 26607	+FBL587	no fruits available					
	PI 614701	+FBL587	no fruits available					
