# Supplementary figures and images for: A Genomic and Transcriptomic Study on the DDT-Resistant Trichoderma hamatum FBL 587: First Genetic Data into Mycoremediation Strategies for DDT-Polluted Sites
Source: Microorganisms. 2021 Aug 7;9(8):1680. doi: 10.3390/microorganisms9081680 (PMC8401308; doi:10.3390/microorganisms9081680)

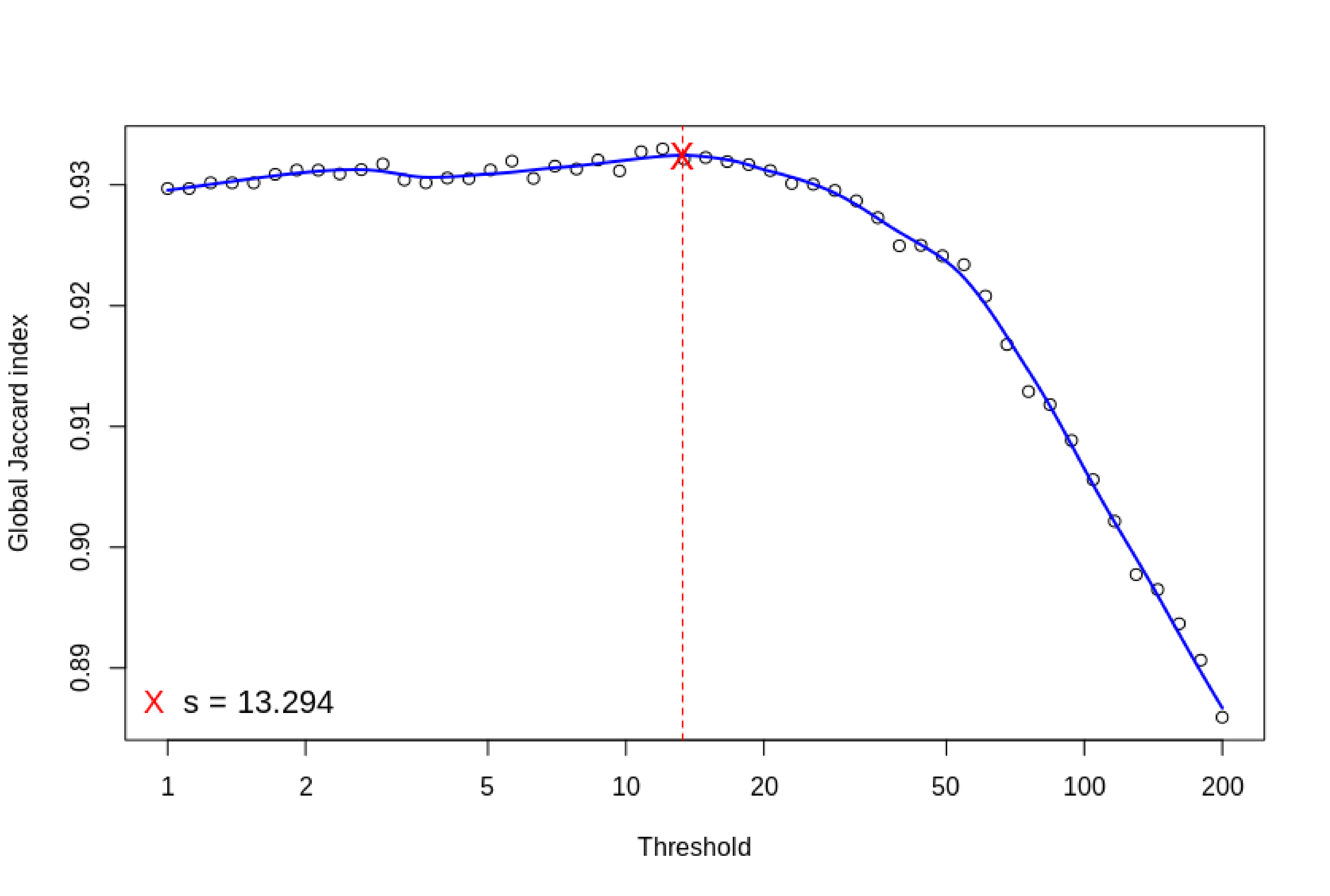

Supplement: Supplementary file 1 [file microorganisms-09-01680-s001.zip › Figure S1.tif]
